# Subarachnoid Hemorrhage Associated with Intratumoral Aneurysm Rupture within a Posterior Fossa Hemangioblastoma: The Importance of Continued Surveillance for Cerebral Vasospasm

**DOI:** 10.7759/cureus.1606

**Published:** 2017-08-24

**Authors:** Connie Ju, Christina H Wright, James Wright, Yifei Duan, Nicholas C Bambakidis

**Affiliations:** 1 Neurological Institute, Case Western School of Medicine; 2 Neurological Institute, University Hospitals Cleveland Medical Center; 3 Neurological Insitute, University Hospitals Cleveland Medical Center

**Keywords:** aneurysms, delayed ischemic neurologic deficit, hemangioblastoma, subarachnoid hemorrhage, vasospasm

## Abstract

Cerebellar hemangioblastomas are rare tumors of the neuraxis. Only seven cases of hemangioblastoma associated with a cerebral aneurysm have been reported. We report a case of a patient who presented with acute onset headache as a result of subarachnoid hemorrhage (SAH) and hydrocephalus. Radiographic workup revealed a hemangioblastoma with an intratumoral aneurysm. Preoperative cerebral angiography was performed for both embolization as well as characterization of the aneurysm. The patient underwent a suboccipital craniotomy for tumor resection. The patient’s postoperative course was unexpectedly complicated by delayed ischemic neurologic deficit secondary to flow-limiting left internal carotid artery vasospasm. We present a case report, review of the literature, and management considerations for patients who present with tumors and an associated ruptured aneurysm.

## Introduction

Hemangioblastomas account for approximately 7 to 10% of tumors arising in the posterior fossa and can develop sporadically or in association with Von Hippel-Lindau disease (VHL). Despite the highly vascularized nature of the tumor, clinical presentation with spontaneous subarachnoid hemorrhage (SAH) is rare [1-2]. We report the third published case of a vermian hemangioblastoma presenting as SAH and provide a review of similar cases with special consideration of peri-operative management and attention to postoperative complications. Although the patient described in our case was initially neurologically stable postoperatively, he experienced a sudden and significant decline on postoperative day nine due to severe left internal carotid artery (ICA) and middle cerebral artery (MCA) flow-limiting vasospasm. This case highlights the importance of vigilant surveillance for delayed ischemic neurologic deficit (DIND) in the setting of SAH irrespective of underlying etiology.

## Case presentation

**Figure 1:**
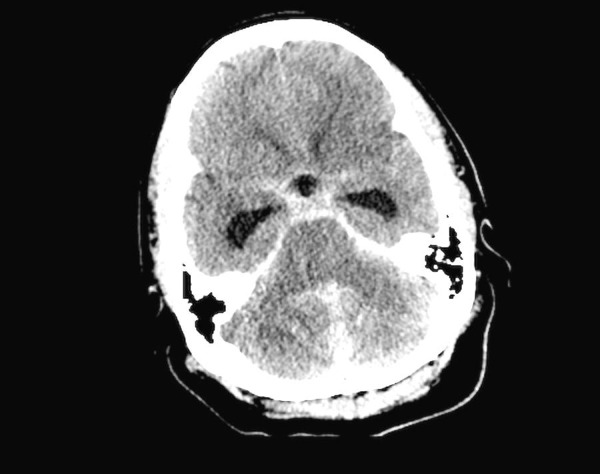
Computed tomography (CT) head: time of admission. Non-contrasted CT of the head showing midline cerebellar intraparenchymal hemorrhage, diffuse subarachnoid hemorrhage, and ventriculomegaly.

**Figure 2:**
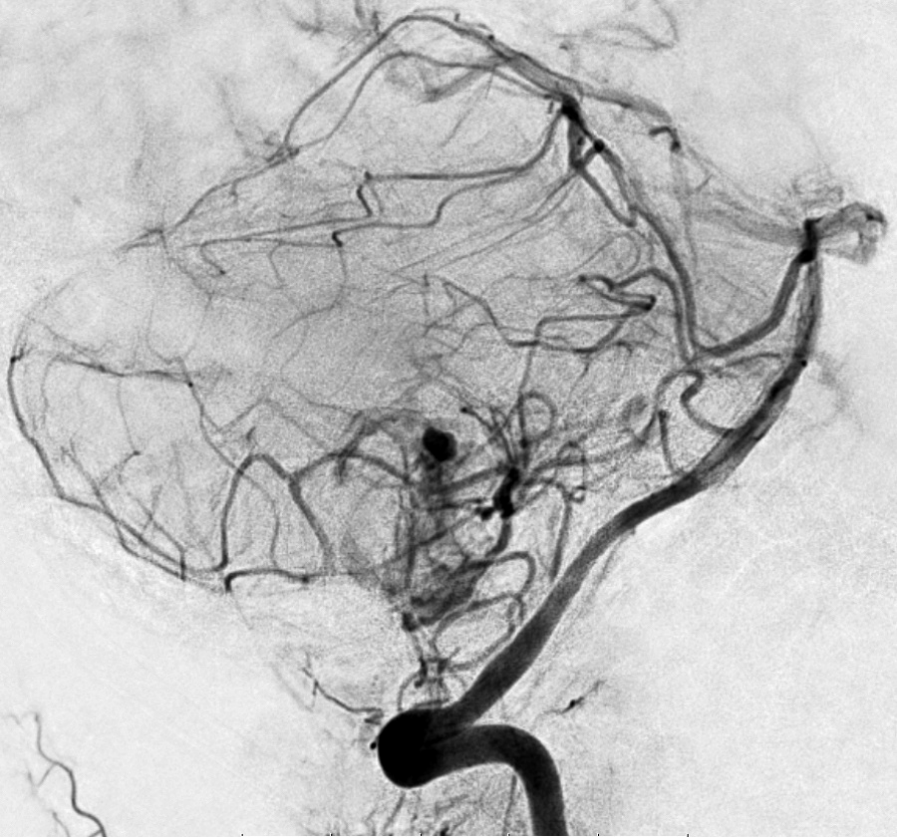
Preoperative angiogram. A left vertebral artery injection angiogram demonstrates tumor blush and intra-tumoral aneurysm.

**Figure 3:**
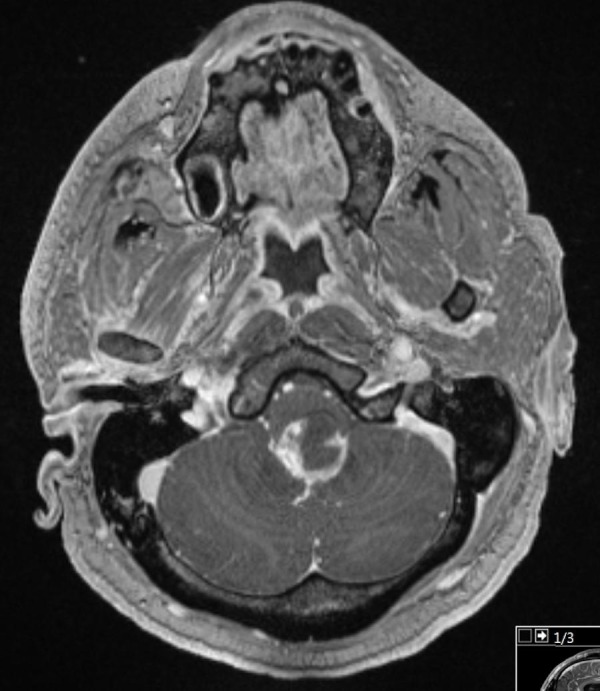
Preoperative magnetic resonance imaging (MRI). Post-gadolinium T1 MRI of the brain showing the enhancing lesion in the midline.

**Figure 4:**
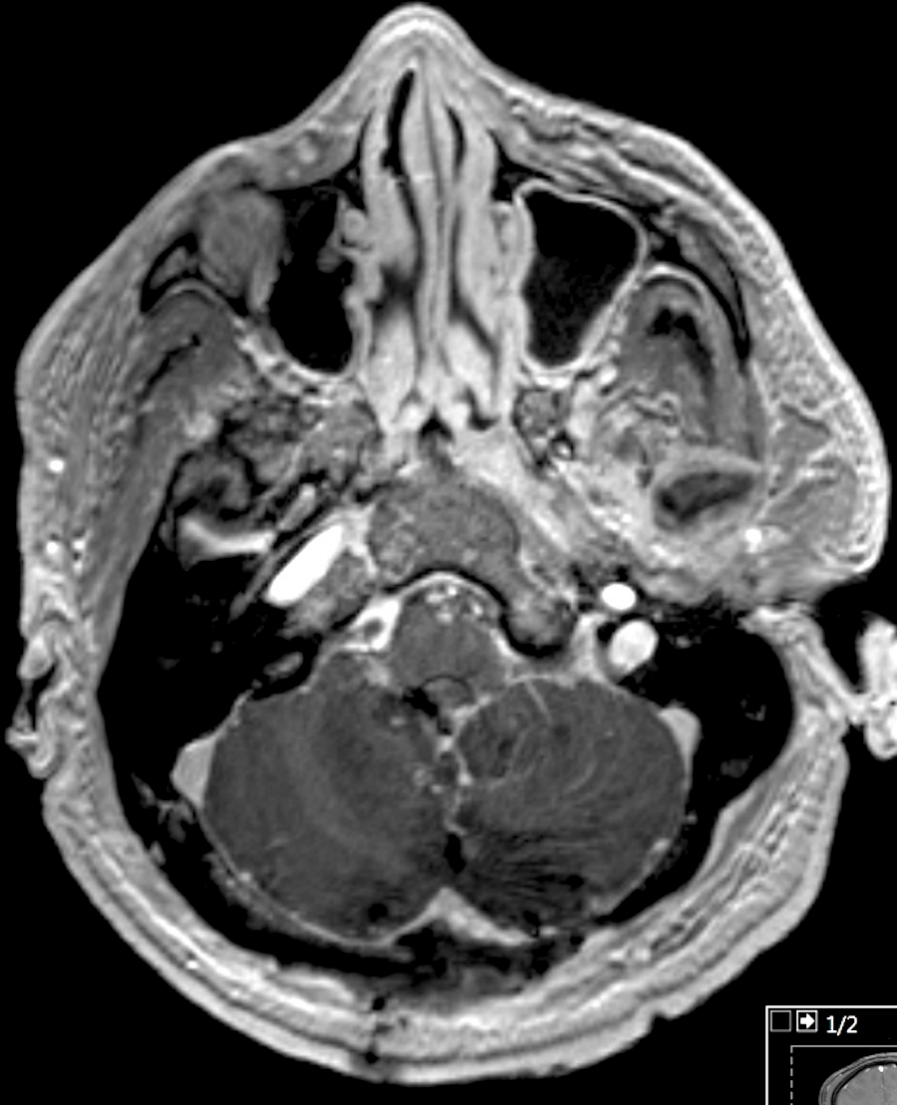
Postoperative magnetic resonance imaging (MRI). Post-gadolinium postoperative MRI demonstrating extent of resection and operative changes.

**Figure 5:**
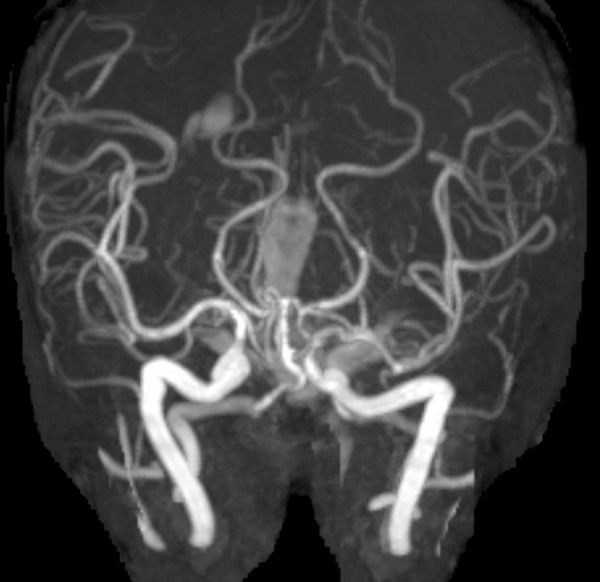
Postoperative magnetic resonance angiography (MRA). Immediate postoperative MRA showing no evidence of vasospasm.

**Figure 6:**
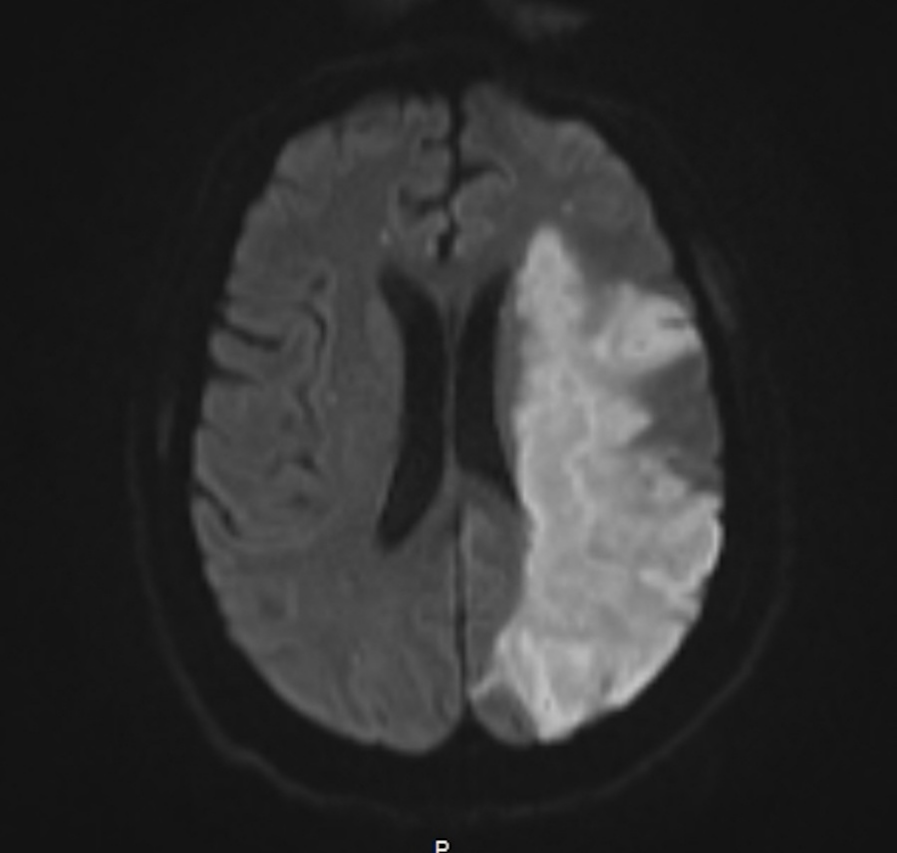
Magnetic resonance imaging (MRI) brain. Delayed MRI demonstrating large left middle cerebral artery (MCA) territory infarction.

**Figure 7:**
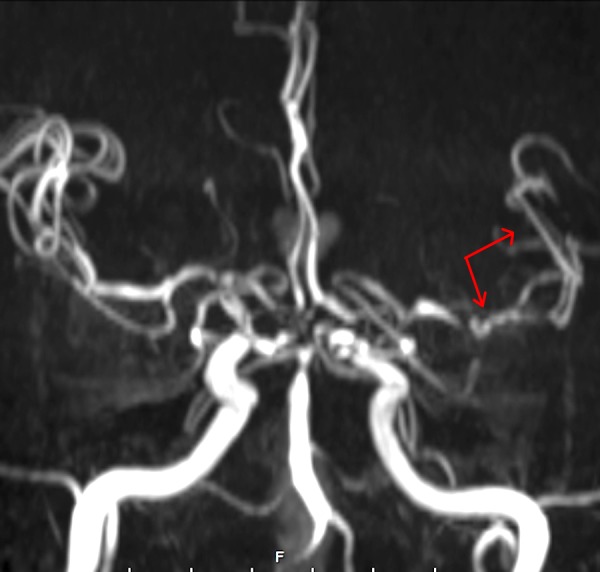
Delayed magnetic resonance angiography (MRA). MRA obtained at the time of neurologic change demonstrated severe left middle cerebral artery (MCA) vasospasm. Note the proximal and distal vessel spasm (red arrows), as compared to the contralateral vessel caliber.

A 70-year-old male presented to the hospital with complaints of severe headache, confusion, and gait instability. He became progressively obtunded in the emergency department. A non-contrast computed tomography (CT) of the head demonstrated diffuse SAH and ventriculomegaly (Figure 1). The patient was intubated and an external ventricular drain (EVD) was placed. He demonstrated mild improvement in alertness after cerebrospinal fluid (CSF) diversion. Cerebral angiography was performed and demonstrated tumor blush in the posterior fossa with an associated intratumoral aneurysm arising from a right posterior inferior cerebellar artery (PICA) branch (Figure 2). Magnetic resonance imaging (MRI) demonstrated a 3 cm enhancing lesion in the area of the cerebellar vermis (Figure 3). In an effort to reduce tumor vascularity and aid in surgical resection, arterial feeders to the tumor and to the aneurysm were embolized with polyvinyl alcohol particles. After embolization, the patient was transferred to the operating room and underwent a suboccipital craniotomy for resection of the mass. Pathology revealed the diagnosis of hemangioblastoma. The initial postoperative course was benign. MRI demonstrated a gross total resection and magnetic resonance angiography (MRA) showed no evidence of aneurysmal remnant (Figures 4, 5). Due to his neurological status and persistence of lower cranial nerve deficits, the patient was unable to be extubated and ultimately required tracheostomy placement. On postoperative day nine and post-admission day 14, the patient experienced a sudden deterioration in his neurologic status. The patient’s motor exam declined from following commands to extensor posturing. Transcranial doppler (TCD) was performed and demonstrated markedly elevated left MCA and ICA velocities of 207 cm/s and 196 cm/s, respectively. Emergent CT obtained at that time was grossly stable when compared to the postoperative MRI. CT and MRI were repeated three days following the patient’s initial decline to work up a persistently poor neurologic exam and revealed significant left MCA territory infarction (Figure 6). MRA at that time demonstrated severe left ICA and MCA flow-limiting vasospasm (Figure 7). The patient was placed on vasopressors in an effort to prevent further ischemic injury. His neurological status remained poor. Ultimately, he was discharged to a long term acute care facility and expired approximately one month after discharge.

## Discussion

Hemangioblastomas are highly vascularized tumors that occur due to sporadic development or as a result of VHL. Sporadic cases represent over 70% of hemangioblastomas, which are most often discovered in the posterior cerebellum or cerebellar vermis [1-2,4]. These tumors are uncommon, comprising only 7-10% of tumors arising in the posterior fossa. The number of cases presenting with SAH is even lower with an incidence of 1% [3-4]. While the true etiology of sporadic hemangioblastomas remains elusive, many studies have proposed molecular culprits and developmental anomalies that result in altered vascular hemodynamics. Somatic mutations or allelic deletion of the VHL tumor suppressor gene on chromosome 3p have been implicated in cases of both sporadic and VHL-related hemangioblastomas, leading to a gain-of-function activity of hypoxia-inducible factor (HIF). Upregulation of vascular elements by HIF, including erythropoietin and vascular endothelial growth factor, may be responsible for the alteration in vasculature and subsequent development of hemangioblastomas [5-7]. In comparison to VHL-related cases, the pathogenesis of sporadic hemangioblastomas may also be strongly driven by regulatory elements encoded in the 3p region other than the VHL tumor suppressor [7].

Presenting neurologic deficits are typically caused by direct tumor compression or tumor hemorrhage. Clinical manifestations depend on the location and nature of the lesion, but common signs and symptoms arising from lesions of the cerebellar region include obstructive hydrocephalus, headache, ataxia, and nausea [2,4]. In this case, this patient presented with rupture of an intratumoral aneurysm, SAH, and hydrocephalus. Many reports describe hemorrhage associated with cerebellar hemangioblastomas, but few have disclosed tumor-associated aneurysms [8-10]. Of these, four cases developed aneurysms of the distal vessel and four developed aneurysms of the feeding artery [10]. The detection of aneurysms occurred incidentally upon angiographic imaging. Only two cases reported aneurysmal rupture during the clinical course [8,10]. See table located in Appendix for the summary of previous cases.

Management of cerebellar hemangioblastomas uniquely encompasses nuances of both tumor and vascular malformation resection. The primary treatment modality is surgical resection while radiation therapy is reserved for unresectable tumors. Preoperative embolization is often completed to reduce bleeding risk and facilitate tumor resection, but some studies found no reduction in operative complications and rather an increased risk of postoperative morbidity and mortality [4]. The decision to pursue embolization in this patient was based on the intention to reduce intraoperative blood loss and facilitate surgical resection of the mass. Of the previously mentioned six documented cases since 1990, three underwent preoperative embolization and all reported successful tumor resection without intraoperative complications. In one case, embolization was considered but not performed [10].

Vasospasm and DIND following tumor resection are rare. Proposed mechanisms of vasospasm following aneurysmal bleeding include smooth muscle constriction secondary to increased calcium release, dysregulation of vasoconstrictors and vasodilators, and vascular remodeling. Vigilant monitoring can prevent significant morbidity and mortality after aneurysmal SAH (aSAH) but typically does not apply to tumor resection. One case reported by Prontera, et al. documented vasospasm of the anterior inferior cerebellar artery 12 days after resection of a medullary hemangioblastoma [10]. Though profuse bleeding was encountered during surgery, no aneurysm was evident. An angiogram had not been performed prior to surgery. Recent reviews report that vasospastic episodes typically occur between three and eight days following cerebellar tumor resection. This observed time to development is similar to that for aSAH, where the risk of vasospasm peaks three to eight days after hemorrhage. In patients presenting with considerable SAH secondary to intra-tumoral aneurysm rupture, extended intensive care unit surveillance is warranted. In cases such as these, the institutional standards for the monitoring and prevention of vasospasm and DIND should be strictly followed.

## Conclusions

Hemangioblastoma presenting with aneurysmal SAH is atypical. Preoperative embolization of the tumor in similar cases is warranted for the reduction of intraoperative bleeding and has been supported with previously described good outcomes. The few similar cases available for review encourage the initiation of an institution’s aSAH management protocol to prevent vasospasm and DIND, although the true risk remains uncharacterized. In the case of neurologic decline in circumstances such as these, it is imperative that early diagnosis and treatment must include vascular imaging and measures to prevent DIND once further hemorrhage has been excluded.

## Appendices

**Table 1:** Review of the prior case reports documenting associated aneurysms with hemangioblastoma. BA: Basilar artery; ICA: Internal carotid artery; AICA: Anterior inferior cerebral artery; PICA: Posterior inferior cerebral artery; SAH: Subarachnoid hemorrhage; POD: Postoperative day; GOS: Glasgow Outcome Scale.

Author	Aneurysm	Presentation	Treatment	Complications	Outcome	Follow-up
Yoshii, et al. (1976)	BA bifurcation Left ICA bifurcation	Tumoral SAH	Surgical resection	---	---	SAH 23 months after tumor resection
Guzman, et al. (1999)	Left distal AICA	Tumoral	Surgical resection & clipping	Intraoperative rupture	Not reported	Not reported
Menovsky, et al. (2002)	Left BA-AICA	Tumoral	Surgical resection & fibrin sealant	None reported	Good	SAH 5 years after surgical resection
Zager, et al. (2002)	Right distal AICA	Tumoral	Embolization & resection	None reported	Good	Not reported
Murai, et al. (2006)	Right ICA	Tumoral	Surgical resection	None reported	Good	Not reported
Seong, et al. (2011)	Left PICA	Tumoral	Embolization & resection	None reported	Not reported	Not reported
Suzuki, et al. (2014)	Left distal PICA	SAH	Embolization & resection	None reported	Not reported	Not reported
Prontera, et al. (2014)	None	Tumor	Resection associated with profuse bleeding	Vasospasm on POD12	Hemiplegic	GOS 3 at 15 months
Present case	Right PICA	SAH	Embolization & resection	Vasospasm on POD 9	Hemiplegic	Deceased at one month
